# Interpreting cranial abdominal coverage after caudal epidural local anesthetic administration in dogs and cats

**DOI:** 10.3389/fvets.2026.1921474

**Published:** 2026-09-09

**Authors:** Shotaro Nagahama

**Affiliations:** 1 JAVA Incorporated Association, Tokyo, Japan; 2 Department of Veterinary Anesthesia, Veterinary Teaching Hospital, Faculty of Applied Biological Sciences, Gifu University, Gifu, Japan

**Keywords:** cats, cranial abdominal coverage, dogs, dose-volume regimen, epidural local anesthetics, functional blockade, injectate distribution, multimodal anesthesia

## Abstract

Caudal epidural local anesthetic administration is used in dogs and cats and may be interpreted as contributing to cranial abdominal coverage, but favorable perioperative outcomes do not identify the cranial extent or completeness of the local-anesthetic block. This conceptual analysis distinguishes anatomical distribution, functional attenuation of predefined stimuli, physiological tolerance, and composite perioperative clinical performance. In feline ovarian surgery, 0.3 ml/kg regimens have produced heterogeneous clinical outcomes, whereas target-oriented CT mapping did not show uniform bilateral T10-T11 foraminal reach even with cumulative iohexol volumes of 0.75–1.0 ml/kg. Differences in injectate, dosing sequence, and endpoint preclude a direct dose-response comparison, but the magnitude of this discrepancy makes consistent complete bilateral coverage after 0.3 ml/kg a less plausible explanation for favorable outcomes. Canine data support prospective testing of a 0.6 ml/kg dose-volume regimen; feline data support comparison of 1.0 ml/kg as an adequacy-oriented candidate with 0.75 ml/kg as a lower comparator. Future studies should aim to connect target-oriented anatomical mapping, procedure-relevant functional testing, physiological assessment, and clinical evaluation using closely matched regimens, with any necessary differences made explicit.

## 1 Introduction

Caudal epidural local anesthetic administration is an established component of small-animal anesthesia and analgesia, particularly for procedures involving the pelvic limbs, perineal region, and caudal abdomen (1). In dogs and cats, lumbosacral or sacrococcygeal access is often used as a single-shot technique unless catheter placement is specified. Because a single-shot injection cannot usually be titrated after injection on the basis of its observed segmental extent, the selected dose-volume regimen becomes a central design variable. Expressing volume in ml/kg normalizes injectate volume to body weight but does not identify the spinal segments, nerve roots, or procedure-specific nociceptive targets intended to be affected (1, 2).

Current veterinary evidence addresses several questions. Anatomical and imaging studies show where an injectate may distribute. Functional tests differ along three independent dimensions: the stimulus applied, the tissue or nociceptive field stimulated, and the response used to define attenuation. Mechanical pressure or pinch, controlled electrical stimulation, and surgical manipulation are different stimuli; skin, muscle, peritoneum, ovary, and other viscera are different targets; and conscious nocifensive behavior, motor responses, and autonomic or hemodynamic changes are different response measures. Anesthetic state, stimulus intensity, and timing further affect interpretation. Clinical studies estimate composite perioperative performance through anesthetic and analgesic requirements, protocol-defined rescue use, recovery, and postoperative pain. These endpoints are informative but not interchangeable.

Target-specific blockade may contribute to clinical success, but clinical success cannot retrospectively establish the extent or completeness of that blockade.

In this analysis, reliable cranial abdominal coverage refers to reproducible bilateral functional attenuation attributable to the epidural local anesthetic at predefined procedure-relevant somatic and visceral targets, sustained for the relevant surgical events. Anatomical target reach, stimulus-specific functional attenuation, physiological tolerance, and composite perioperative clinical performance are treated as distinct but related evidentiary domains.

The analysis focuses on caudal single-shot epidural local anesthetic administration. More cranial neuraxial or non-neuraxial regional techniques may be appropriate for selected procedures, but comparative evaluation of those approaches is outside the scope of this analysis. Epidural opioids, alpha-2 agonists, and other neuraxial adjuncts are outside its primary scope, except when they form part of a clinical regimen whose composite outcome is being interpreted.

## 2 Evidentiary domains and the claims they support

Four evidentiary domains are distinguished. Anatomical distribution refers to dye, contrast, or imaging evidence that injectate reached a vertebral level, intervertebral foramen, nerve-root region, or other predefined anatomical target. Stimulus-specific functional attenuation refers to a reproducible reduction in a predefined response to a defined stimulus in a specified tissue or nociceptive field under specified conditions. Physiological tolerance refers to the respiratory, hemodynamic, and motor consequences of the regimen, including the need for assisted ventilation or cardiovascular support. Composite perioperative clinical performance refers to anesthetic and analgesic requirements, protocol-defined rescue use, recovery, and postoperative pain within the anesthetic plan as a whole.

The four domains are not ordered from weak to strong evidence. Each can be studied rigorously, but each supports a different type of claim. A distribution study can provide strong evidence of anatomical reach without testing neural function, whereas a clinical trial can establish incremental benefit without identifying the segmental mechanism that produced it. Within the functional domain, a result supports only the tested combination of stimulus method, stimulated tissue, response criterion, anesthetic state, stimulus intensity, and timing; it cannot be assumed to represent another combination.

Studies were selected purposively because they illustrate one or more of the four evidence types, measure more than one type within the same design, or directly inform the proposed canine and feline comparisons.

Results from different domains can be related only when species and model, access and delivery, injectate regimen, position and timing, anatomical target, stimulus, and response criterion are sufficiently aligned. Table 1 summarizes what each selected study measured, what it showed, and what remains unresolved.

**Table 1:** Selected studies informing the evaluation of cranial abdominal coverage after epidural local anesthetic administration.

Study	Population/model and design	Epidural strategy	Endpoint(s) relevant to this analysis	Finding relevant to cranial coverage	Question left unresolved
Freire et al. (5)	20 healthy adult female mixed-breed dogs; prospective randomized four-group experiment (*n* = 5/group)	Lumbosacral catheter advanced 5 cm cranially; dogs standing; 0.25% bupivacaine plus methylene blue at 0.2, 0.4, 0.6, or 0.8 ml/kg	Mechanical skin pinch; dorsal skin, tail, and pelvic-limb sites; conscious nocifensive response; motor scores; postmortem dural staining	Dorsal cutaneous block increased through 0.6 ml/kg. Staining reached the cervical region in some dogs at 0.4 ml/kg and all dogs at 0.6 ml/kg; gross staining and cutaneous response did not scale identically.	No procedure-specific cranial abdominal somatic or visceral test. Total bupivacaine dose increased with volume; catheter advancement, standing position, and a cranial endpoint ceiling limit extrapolation
Aso et al. (6)	6 healthy Beagles; prospective randomized three-period crossover experiment	Co1-Co2 injection; equal volumes of 1% ropivacaine and iotrolan 240 at 0.1, 0.2, or 0.3 ml/kg	Controlled electrical stimulation at predefined nonvisceral body sites; HR/MAP response; CT distribution and leakage; motor function	Increasing volume extended the region in which electrical stimulation failed to elicit a 15% or greater HR/MAP response, whereas CT spread did not differ significantly; contrast laterality and response laterality were discordant.	Electrically evoked autonomic responses at nonvisceral sites are not equivalent to cranial abdominal surgical responses. Contrast admixture and foraminal leakage complicate extrapolation
Cima et al. (7)	10 analyzed living female dogs and 10 thawed canine cadavers; prospective comparative anatomical study	Fluoroscopy-guided L1-L2 injection of 0.2 ml/kg 0.25% bupivacaine plus iohexol	Fluoroscopic cranial distribution	Distribution was more extensive in living dogs than in thawed cadavers, indicating that cadaveric models may underestimate *in vivo* distribution	More cranial access rather than a caudal single shot; no procedure-specific functional test or individual distribution-to-response analysis
Freitag et al. (8)	6 healthy female Beagles; prospective randomized crossover experiment	Lumbosacral catheter; 0.25% bupivacaine plus iopamidol; BW dosing at 0.2 ml/kg (1.81 ± 0.21 ml) vs. OCL-based dosing (3.29 ± 0.74 ml); sternal recumbency	CT rostral distribution; postanesthetic sensory and motor assessments; cardiorespiratory variables	OCL-based dosing delivered 1.82-fold more volume and increased mean CT reach from 19 ± 2 to 22 ± 2 vertebrae. No differences were detected in the postanesthetic sensory, motor, or cardiorespiratory outcomes; farther mean spread did not establish a functional advantage or improved predictive precision	Six homogeneous small dogs; no surgical or cranial abdominal target. The SD of CT reach was 2 vertebrae under both methods, and the study was not designed to test whether OCL reduced between-animal variability or prediction error after calibration to a common target. Catheter use and contrast admixture also limit extrapolation
Freitag et al. (9)	22 canine cadavers; controlled comparative cadaver study	Lumbosacral catheter; mixture of iopamidol and new methylene blue; iopamidol assessed by early CT and dye by later dissection	Early CT distribution of iopamidol; later anatomical distribution of new methylene blue	New methylene blue observed at later dissection extended farther cranially than iopamidol on earlier CT, indicating that these anatomical endpoints are not interchangeable	Marker, assessment method, and assessment time differed, so the discrepancy cannot be attributed to marker alone. Neither endpoint measured neural function
dos-Santos et al. (10)	50 feral female cats undergoing elective ovariectomy; prospective randomized clinical comparison (*n* = 25/group)	Sacrococcygeal 0.25% bupivacaine 0.3 ml/kg vs. bilateral quadratus lumborum block with 0.25% bupivacaine 0.4 ml/kg; multimodal general anesthesia	Intraoperative rescue use and hemodynamics; extubation; postoperative pain classification and motor block	Only 3/25 epidural cats required intraoperative rescue, and postoperative pain classifications were similar to quadratus lumborum block, indicating favorable composite performance.	No anatomical target mapping or segmental functional test. Favorable composite performance cannot establish bilateral T10-T11 reach or ovarian target blockade; the active
				Hypotension, extubation delay, and motor block were more frequent after epidural administration	comparator and multimodal protocol also contribute to the observed outcome
Zahra et al. (11)	36 female cats undergoing elective ovariohysterectomy; prospective randomized three-group clinical trial (*n* = 12/group)	Sacrococcygeal or lumbosacral 0.33% levobupivacaine 0.3 ml/kg vs. 0.9% saline at the same volume; multimodal general anesthesia	End-tidal isoflurane concentration; intraoperative fentanyl requirements; cardiorespiratory variables; postoperative pain, rescue morphine, and time to standing	Both levobupivacaine groups had modestly lower isoflurane and fentanyl requirements than saline, but postoperative pain and rescue analgesia did not differ. The same 0.3 ml/kg nominal volume therefore produced limited rather than uniformly strong clinical effects	No injectate mapping or segmental functional test. The findings are compatible with incomplete procedure-relevant coverage but do not establish its anatomical or functional extent
Wong et al. (12)	7 healthy intact female domestic shorthair cats; prospective within-animal CT distribution study	Sacrococcygeal stimulating needle; iohexol 44 mg/mL in four sequential 0.25 mL/kg boluses with CT after each bolus (cumulative 0.25-1.0 mL/kg)	Left- and right-sided cranial CT distribution; bilateral reach to the T10-T11 intervertebral foramina	Bilateral T10-T11 reach occurred in 3/7 cats at 0.75 ml/kg and in 6/7 by 1.0 ml/kg. At 1.0 ml/kg, 1 cat remained short of the target	Iohexol rather than local anesthetic; sequential rather than single-bolus administration; no functional blockade or clinical analgesic endpoint. The study supports 1.0 ml/kg as an adequacy-oriented candidate and 0.75 ml/kg as a lower comparator, not as validated regimens
Dias et al. (14)	6 healthy adult mixed-breed dogs; prospective randomized crossover experiment	Lumbosacral 0.25% bupivacaine at 0.2 or 0.4 ml/kg during spontaneous ventilation at 1.3 MAC isoflurane	Invasive hemodynamics; ventilation; blood gases; clinical signs through 90 min	The 0.4 ml/kg regimen produced greater mean arterial pressure depression, more hypotension, and transient hypoventilation	No functional coverage endpoint and no 0.6 ml/kg regimen. Constant isoflurane concentration and experimental conditions limit direct clinical generalization
Parreño et al. (15)	210 dogs undergoing abdominal surgery; single-center retrospective observational study with nonconcurrent cohorts (OCL *n* = 110; BW *n* = 100)	L7-S1 levobupivacaine with morphine or buprenorphine; total volume calculated by OCL or BW	Post-epidural hypotension; administered volume; multivariable and machine-learning risk models	Mean volume was 0.448 vs. 0.241 ml/kg and hypotension occurred in 49.1% vs. 33% of dogs with OCL vs. BW calculation	Retrospective nonconcurrent cohorts, combined drug regimens, and multiple perioperative differences preclude a causal volume-only inference; no coverage endpoint

Endpoints and findings are limited to those relevant to the present conceptual analysis. The selected studies address different questions relevant to cranial coverage; the table is not a comprehensive evidence inventory or an overall quality ranking. BW, body-weight-based calculation; CT, computed tomography; HR, heart rate; MAP, mean arterial pressure; OCL, occipito-coccygeal-length-based calculation.

## 3 Limits of indirect evidence for cranial abdominal coverage

### 3.1 Anatomical distribution does not establish functional blockade

Anatomical studies establish where an injectate can distribute and identify factors that shape that distribution. Classical cryomicrotome studies of the human epidural space showed that epidural anatomy and solution distribution are nonuniform and are influenced by fat, connective tissue, vascular structures, root sleeves, fascial barriers, and pressure relationships (3, 4). Although these studies are not species-specific evidence for dogs or cats, they provide an anatomical basis for interpreting visible epidural solution at a level of interest as evidence of reach rather than a direct measurement of neural exposure or neural block.

Functional blockade additionally requires sufficient local anesthetic exposure at the relevant neural structures, in both concentration and duration. At the cranial margin of a distribution pattern, the mass, concentration, and duration of drug exposure may be lower than at segments closer to the injection site. Contrast or dye at a vertebral level or intervertebral foramen therefore cannot be assumed to demonstrate effective nerve-root blockade. Meningeal diffusion, dilution within cerebrospinal-fluid sleeves, and subsequent access to neural tissue may also influence block, but the quantitative relationship between these processes and imaging appearance remains incompletely defined in dogs and cats.

Selected studies show that imaging and tested functional responses can diverge even within the same experiment (Table 1). Freire et al. found cervical staining in some dogs at 0.4 ml/kg while the mean dorsal cutaneous block increased further at 0.6 ml/kg; the dye endpoint approached its cranial ceiling, and the tested response was cutaneous rather than abdominal visceral (5). Aso et al. paired CT distribution with electrically evoked autonomic responses and found discordance between imaging and response laterality; the tested sites were nonvisceral (6).

Apparent distribution also depends on model and measurement. Cima et al. observed more extensive spread in living dogs than in thawed cadavers (7). Freitag et al. found farther mean CT spread with OCL-based than BW-based dosing, but the study did not establish a functional advantage or lower between-animal variability (8); in a related cadaver experiment, later dissection showed more cranial methylene-blue distribution than earlier CT showed for iopamidol (9).

Together, these studies show that visible spread varies with the experimental model and assessment method and may not match the tested functional response. Details of study design and unresolved questions are summarized in Table 1.

### 3.2 Clinical benefit does not establish the extent of cranial blockade

Clinical findings at 0.3 ml/kg are heterogeneous (Table 1). In dos-Santos et al., sacrococcygeal bupivacaine was associated with low intraoperative rescue use and postoperative pain classifications similar to quadratus lumborum block within a multimodal protocol (10). In Zahra et al., the same nominal volume produced only modest intraoperative sparing and no postoperative analgesic benefit (11). Thus, 0.3 ml/kg does not define a uniform clinical profile or reveal the anatomical extent of blockade.

Wong et al. show how limited the anatomical inference from these clinical findings remains. Bilateral T10-T11 reach occurred in 3 of 7 cats at 0.75 ml/kg and in 6 of 7 by 1.0 ml/kg, with 1 cat still short of the target (12). Differences in injectate, administration sequence, timing, and endpoint preclude a direct dose-response comparison. Nevertheless, the 2.5- to 3.3-fold volume discrepancy makes it difficult to assume that favorable outcomes at 0.3 ml/kg usually reflected consistent, complete bilateral coverage. These studies do not show that 0.3 ml/kg failed in any individual cat. Favorable outcomes could still reflect complete coverage in some cats, partial but useful blockade, or substantial effects of the other anesthetic and analgesic interventions.

## 4 Candidate caudal dose-volume regimens

### 4.1 Dogs: prospective evaluation of 0.6 ml/kg

Canine data provide an empirical anchor for selecting a candidate regimen for prospective testing. In the Freire et al. experiment, mean numbers of dorsally tested dermatomes blocked at 15–20 min were 5.0, 14.2, 20.2, and 21.0 for the 0.2, 0.4, 0.6, and 0.8 ml/kg regimens, respectively; T1 was scored as 20 and the cervical region as 21 (5). Dye reached the cervical vertebral region in all dogs at 0.6 ml/kg. These findings support prospective evaluation of a 0.6 ml/kg dose-volume regimen for extensive cranial coverage in dogs.

The experiment does not identify a minimum effective volume, an optimal regimen, a routinely acceptable clinical dose, or a volume-specific causal threshold. Because concentration was fixed at 0.25%, total bupivacaine dose increased with volume. The catheter was advanced 5 cm cranially and was found ventral in all dogs, the dogs were kept standing, the cranial response scale had a ceiling, and the functional endpoint was dorsal cutaneous nociception rather than cranial abdominal somatic or visceral input. The 0.6 ml/kg regimen is therefore an evidence-anchored candidate for direct testing.

### 4.2 Cats: prospective comparison of 1.0 and 0.75 ml/kg

Wong et al. identified 0.75 ml/kg as the first tested cumulative volume at which bilateral T10-T11 reach occurred, but this endpoint was achieved in only 3 of 7 cats. By 1.0 ml/kg, bilateral reach had occurred in 6 cats, although it remained incomplete in 1 (12). For a study primarily designed to test target-specific adequacy, 1.0 ml/kg therefore represents the more defensible primary candidate total volume, whereas 0.75 ml/kg is a useful lower comparator for examining the trade-off between target reach and excessive cranial distribution. Neither value defines a minimum effective volume or guarantees uniform coverage. At 0.75 ml/kg, iohexol reached T4-T5 at least unilaterally in 1 cat; after 1.0 ml/kg, it reached that level in 3 additional cats (12). The sequential iohexol protocol also cannot establish that a single injection of local anesthetic at either volume would produce the same anatomical or functional result.

A later randomized clinical trial used a 0.75 ml/kg total sacrococcygeal volume containing preservative-free morphine 0.1 mg/kg and ropivacaine 0.93 mg/kg after ovariohysterectomy and found lower early pain scores and less rescue robenacoxib use than in controls (13). Because the injection was administered after surgery and combined an epidural opioid with a local anesthetic, the trial evaluated postoperative analgesic performance rather than intraoperative local-anesthetic-specific cranial coverage. It therefore does not resolve whether 0.75 ml/kg is adequate for the target defined by Wong et al.

### 4.3 Concurrent evaluation of physiological tolerance

Coverage and physiological tolerance need concurrent evaluation. The cranial distribution observed by Wong et al. identifies a potential for extension beyond the ovarian target, but it does not establish a hemodynamic or respiratory effect (12). In six isoflurane-anesthetized dogs, 0.4 ml/kg of 0.25% lumbosacral bupivacaine produced greater mean arterial pressure depression, more hypotension, and transient hypoventilation than 0.2 ml/kg (14). In a retrospective cohort of 210 dogs undergoing abdominal surgery with lumbosacral levobupivacaine-opioid regimens, occipito-coccygeal-length-based calculation produced a greater mean volume and was associated with more post-epidural hypotension than body-weight calculation (15). The association does not isolate volume causally because the cohorts were nonconcurrent and multiple perioperative factors differed. Severe hypoventilation and cardiovascular depression after 0.6 ml/kg bupivacaine in two dogs provide a case-level safety signal rather than an incidence estimate (16). Freire et al. also observed prostration and respiratory depression in many animals receiving larger regimens, without systematic quantification (5).

Prospective evaluation of an adequacy-oriented regimen should prespecify monitoring, ventilation criteria, hemodynamic rescue thresholds, and stopping rules. A feline comparison of 1.0 and 0.75 ml/kg would be most informative if it measured bilateral target reach, distribution into the cranial thoracic region, procedure-relevant functional attenuation, ventilation, hemodynamics, and motor effects under the same local-anesthetic regimen. Physiological acceptability will also depend on patient respiratory reserve, airway control, anesthetic depth, monitoring, and immediate access to ventilatory and cardiovascular support.

## 5 Discussion

### 5.1 Matching claims to measured endpoints

Four questions can guide interpretation of a study or clinical report. First, what anatomical and nociceptive target is being claimed? Ventral abdominal wall incision, parietal peritoneal manipulation, ovarian traction, and cranial visceral traction do not necessarily represent the same field. Second, which evidentiary domain was measured: anatomical reach, stimulus-specific functional attenuation, physiological tolerance, or composite perioperative performance? Third, which mechanisms remain compatible with the observed outcome? Fourth, if evidence from different domains is being combined, are species, access route, injectate regimen, target, timing, stimulus, and response criterion sufficiently aligned?

Two questions are particularly useful when anatomical reach and functional adequacy are discussed together: Did the injectate reach the predefined bilateral anatomical targets? Did the local anesthetic produce reproducible attenuation of the predefined procedure-relevant stimuli for sufficient duration? Together, these questions convert a general coverage claim into two independently testable endpoints.

### 5.2 Linking anatomical, functional, physiological, and clinical evidence

Future work can proceed in stages rather than measuring all four outcomes in one trial. An anatomical study can first define bilateral reach and variability. A living-animal study can then test the same or a closely matched regimen against a predefined functional stimulus. At the functional stage, procedure-relevant noxious events can be prespecified while appropriate background anesthesia and rescue protocols remain available. Separate studies can assess physiological effects and clinical benefit. Table 1 shows that existing studies cover individual stages, but no linked series of methodologically comparable studies has yet validated cranial abdominal coverage. Any differences between stages should be stated explicitly.

### 5.3 Selecting comparators for candidate regimens

Candidate comparisons should follow the question being tested. For dogs, the comparator for 0.6 ml/kg should be prespecified according to whether the study is intended to compare the candidate with a regimen reflecting current caudal practice, an adjacent lower dose-volume regimen, or a dose-matched alternative. In cats, 1.0 ml/kg can serve as the primary adequacy-oriented candidate and 0.75 ml/kg as the lower comparator.

### 5.4 Distinguishing dose-calculation models from volume-effect studies

Comparisons of BW- and OCL-based calculations ask whether one patient-size descriptor predicts achieved cranial distribution more consistently across animals. The potential advantage of OCL would be lower animal-to-animal variation, not simply a larger calculated volume or farther mean spread. BW and OCL formulas can yield different volumes (2), and OCL-based dosing increased mean CT spread in the six-dog crossover study; however, the reported SD was 2 vertebrae under both methods (8). Future studies should calibrate each formula to the same anatomical target and compare the remaining variation. If OCL is more consistent, a target-specific coefficient can then be derived and tested for functional blockade and physiological tolerance.

This question is distinct from whether volume itself changes epidural spread or blockade. To isolate a volume effect, the study must state which factor is held constant. Dose-matched regimens can compare different volumes and concentrations at the same total local-anesthetic dose; complementary comparisons at a fixed volume or fixed concentration can help distinguish effects of volume, concentration, total dose, or their combined effects.

### 5.5 Limitations

Because the literature was selected purposively rather than systematically, relevant reports may have been missed. Among the studies reviewed, none followed the same or a closely matched local-anesthetic regimen from bilateral target mapping through functional testing, physiological assessment, and clinical outcomes. Procedure-specific somatic and visceral targets also remain incompletely mapped for several abdominal operations in dogs and cats.

## 6 Conclusion

In dogs, 0.6 ml/kg is the proposed candidate for prospective evaluation; in cats, the proposed comparison is 1.0 vs. 0.75 ml/kg. In both species, bilateral target reach, procedure-relevant functional attenuation, physiological tolerance, and overall clinical performance should be assessed separately. These volumes are research candidates, not clinical dosing recommendations.

## References

[1] Valverde A. Epidural analgesia and anesthesia in dogs and cats. Vet Clin North Am Small Anim Pract. (2008) 38:1205–30. doi: 10.1016/j.cvsm.2008.06.004 18954681

[2] Valverde A Skelding A. Comparison of calculated lumbosacral epidural volumes of injectate using a dose regimen based on body weight versus length of the vertebral column in dogs. Vet Anaesth Analg. (2019) 46:135–40. doi: 10.1016/j.vaa.2018.10.002 30509673

[3] Hogan QH. Lumbar epidural anatomy. A new look by cryomicrotome section. Anesthesiology. (1991) 75:767–75. doi: 10.1097/00000542-199111000-00007 1952201

[4] Hogan Q. Distribution of solution in the epidural space: examination by cryomicrotome section. Reg Anesth Pain Med. (2002) 27:150–6. doi: 10.1053/rapm.2002.29748 11915061

[5] Freire CD Torres MLA Fantoni DT Cavalcanti RL Noel-Morgan J. Bupivacaine 0.25% and methylene blue spread with epidural anesthesia in dog. Vet Anaesth Analg. (2010) 37:63–9. doi: 10.1111/j.1467-2995.2009.00493.x 20017821

[6] Aso R Itami T Hashimoto M Hori A Wei Y Chen I-Y . Evaluation of drug distribution, sensory and motor blockade regions in canine coccygeal epidural anesthesia. J Vet Med Sci. (2026) 88:257–65. doi: 10.1292/jvms.25-0239 41354431 PMC12887078

[7] Cima DS Credie L de FGA Futema F Luna SPL. Lumbar epidural: anatomical and clinical study in dogs submitted to ovariohysterectomy. Front Vet Sci. (2020) 7:527812. doi: 10.3389/fvets.2020.527812 33240944 PMC7669829

[8] Freitag FAV Valverde A Jensen M Sanchez A Gomez DE Bailey C. Comparison of rostral spread of lumbosacral epidural volume calculated by body weight or length of the vertebral column in small-sized anesthetized dogs. Can J Vet Res. (2023) 87:208–16. 37397637 PMC10291696

[9] Freitag FAV Valverde A Jensen M Sanchez A Gomez DE Bailey C. Rostral spread of lumbosacral epidural volumes of dye and contrast medium calculated using body weight or length of the vertebral column in dog cadavers. Can J Vet Res. (2023) 87:217–23. 37397633 PMC10291695

[10] Dos-Santos JD Ginja M Martins J Cabral P Alves-Pimenta S Ribeiro L . Comparison between bilateral ultrasound-guided quadratus lumborum block and sacrococcygeal epidural in cats undergoing ovariectomy. Vet Sci. (2024) 11:25. doi: 10.3390/vetsci11010025 38250931 PMC10819764

[11] Zahra JOL Segatto CZ Zanelli GR Bruno TDS Nicácio GM Giuffrida R . A comparison of intra and postoperative analgesic effects of sacrococcygeal and lumbosacral epidural levobupivacaine in cats undergoing ovariohysterectomy. J Vet Med Sci. (2023) 85:1172–9. doi: 10.1292/jvms.23-0114 37793832 PMC10686773

[12] Wong S Boesch JM Parry S Gleed RD Martin-Flores M Porter I. Assessment of the epidural distribution of sequential boluses of iohexol injected at the sacrococcygeal space in anesthetized cats. Am J Vet Res. (2025) 86:1–9. doi: 10.2460/ajvr.25.03.0107 40628298

[13] Boesch JM Wong S Tobin K Parry S Muto R Whittington M . Sacrococcygeal epidural injection of morphine and ropivacaine provides analgesia after feline ovariohysterectomy. Am J Vet Res. (2026) 87:1–9. doi: 10.2460/ajvr.25.12.0436 41791227

[14] Dias RSG Soares JHN Castro DDSE Gress MAK de A Machado ML Otero PE . Cardiovascular and respiratory effects of lumbosacral epidural bupivacaine in isoflurane-anesthetized dogs: The effects of two volumes of 0.25% solution. PLoS ONE. (2018) 13:e0195867. doi: 10.1371/journal.pone.0195867 29668768 PMC5906007

[15] Parreño CM Giles D Corletto F. Machine learning analysis of factors contributing to hypotension after lumbosacral epidural anaesthesia in dogs undergoing abdominal surgery. Sci Rep. (2025) 15:18009. doi: 10.1038/s41598-025-01991-3 40410265 PMC12102333

[16] Castro DS Soares JHN Gress MAKA Otero PE Marostica E Ascoli FO. Hypoventilation exacerbates the cardiovascular depression caused by a high volume of lumbosacral epidural bupivacaine in two isoflurane-anesthetized dogs. Vet Anaesth Analg. (2016) 43:235–7. doi: 10.1111/vaa.12320 26577992

